# Artificial Intelligence in Adult Congenital Heart Disease: Diagnostic and Therapeutic Applications and Future Directions

**DOI:** 10.31083/RCM41523

**Published:** 2025-08-28

**Authors:** Ibrahim Antoun, Ali Nizam, Armia Ebeid, Mariya Rajesh, Ahmed Abdelrazik, Mahmoud Eldesouky, Kaung Myat Thu, Joseph Barker, Georgia R Layton, Mustafa Zakkar, Mokhtar Ibrahim, Kassem Safwan, Radek M Dibek, Riyaz Somani, G. André Ng, Aiden Bolger

**Affiliations:** ^1^Department of Cardiology, University Hospitals of Leicester NHS Trust, Glenfield Hospital, LE3 9QP Leicester, UK; ^2^Department of Cardiovascular Sciences, Clinical Science Wing, University of Leicester, Glenfield Hospital, LE3 9QP Leicester, UK; ^3^Department of Acute Medicine, Fiona Stanley Hospital, Perth, WA 6150, Australia; ^4^National Heart and Lung Institute, Imperial College London, SW3 6LY London, UK; ^5^Department of Cardiac Surgery, University Hospitals of Leicester NHS Trust, Glenfield Hospital, LE3 9QP Leicester, UK; ^6^Department of Research, Leicester British Heart Foundation Centre of Research Excellence, LE3 9QP Leicester, UK; ^7^National Institute for Health Research Leicester Research Biomedical Centre, LE3 9QP Leicester, UK

**Keywords:** congenital heart disease, artificial intelligence, machine learning, ECG, risk stratification, remote monitoring, personalised medicine

## Abstract

Adult congenital heart disease (ACHD) constitutes a heterogeneous and expanding patient cohort with distinctive diagnostic and management challenges. Conventional detection methods are ineffective at reflecting lesion heterogeneity and the variability in risk profiles. Artificial intelligence (AI), including machine learning (ML) and deep learning (DL) models, has revolutionized the potential for improving diagnosis, risk stratification, and personalized care across the ACHD spectrum. This narrative review discusses the current and future applications of AI in ACHD, including imaging interpretation, electrocardiographic analysis, risk stratification, procedural planning, and long-term care management. AI has been demonstrated as being highly accurate in congenital anomaly detection by various imaging modalities, automating measurement, and improving diagnostic consistency. Moreover, AI has been utilized in electrocardiography to detect previously undetected defects and estimate arrhythmia risk. Risk-prediction models based on clinical and imaging information can estimate stroke, heart failure, and sudden cardiac death as outcomes, thereby informing personalized therapy choices. AI also contributes to surgery and interventional planning through three-dimensional (3D) modelling and image fusion, while AI-powered remote monitoring tools enable the detection of early signals of clinical deterioration. While these insights are encouraging, limitations in data availability, algorithmic bias, a lack of prospective validation, and integration issues remain to be addressed. Ethical considerations of transparency, privacy, and responsibility should also be highlighted. Thus, future initiatives should prioritize data sharing, explainability, and clinician training to facilitate the secure and effective use of AI. The appropriate integration of AI can enhance decision-making, improve efficiency, and deliver individualized, high-quality care to ACHD patients.

## 1. Introduction

Cardiovascular disease is becoming a healthcare challenge, especially in the developing world [1, 2, 3, 4]. Adult congenital heart disease (ACHD) refers to the growing number of adults living with congenital heart defects (CHD)—the most common type of birth anomaly, affecting nearly 1% of all live births [5]. Thanks to major advancements in paediatric cardiology and cardiac surgery, around 97% of children born with CHD now live into adulthood. In the United States, there are over 1.4 million adults living with CHD, surpassing the number of paediatric patients. On a global scale, improved survival rates and population growth have pushed the number of people living with CHD to nearly 12 million as of 2017 [6]. However, this success has introduced new challenges. ACHD patients often present with complex cardiac anatomies—frequently modified by prior surgeries—and may also develop acquired comorbidities as they age. These complexities make management difficult, requiring lifelong specialised care and careful risk assessment. Yet, traditional clinical decision-making tools often fall short due to the wide variability in lesions and outcomes.

Clinicians utilise established protocols, imaging studies, and risk-scoring systems to inform their care. Still, these conventional methods may not fully capture the individualised and nuanced realities of ACHD. In this context, artificial intelligence (AI)—especially machine learning (ML) and deep learning (DL)—has emerged as a promising solution. These technologies excel at recognising patterns within large datasets and have shown strong potential in various areas of healthcare [7].

In the ACHD setting, AI can help integrate multimodal data—like imaging, electrocardiograms (ECGs), patient history, and genetics—to support complex diagnoses, predict long-term outcomes, and personalise treatment strategies. Yet, despite these possibilities, AI remains underutilised in day-to-day ACHD practice. Recognising this gap, recent expert reviews have urged a focused effort to adopt AI in ACHD care to improve diagnosis, outcome prediction, and treatment planning [8].

While recent reviews and meta-analyses (summarised in Table 1, Ref. [9, 10, 11]) have highlighted the promise of AI in CHD, several methodological gaps remain. Notably, most included studies are based on retrospective data with small to moderate sample sizes and lack external validation across diverse populations. For instance, the systematic review by Mohammadi *et al*. [9] reported high area under the curve (AUC) values for AI-predicted surgical outcomes; however, few studies included multicenter data or prospective evaluation, which limits generalizability. Similarly, the review by Jone *et al*. [10] offered a valuable call-to-action. However, it did not perform a structured quality assessment of included models, and many cited applications were still at the proof-of-concept stage. Furthermore, several reviews combine paediatric and ACHD populations, making it difficult to extract insights specifically relevant to ACHD care. Our review builds upon this literature by focusing on real-world applicability, clinical integration, and prospective translation of AI tools in ACHD, and by outlining a roadmap for future research directions with a focus on validation, explainability, and ethical deployment. We also examine the real-world impact of AI adoption in ACHD care, including expected benefits, limitations, clinician buy-in, and regulatory considerations. Lastly, we outline future directions and key research gaps that must be addressed to fully harness AI’s potential to improve outcomes for ACHD patients.

**Table 1.** **Recent review articles and meta-analyses on artificial intelligence in congenital heart disease**.

Reference (year)	Focus area	Scope	Key findings/gaps
Trayanova *et al*., 2021 [11]	ML in Arrhythmias & Electrophysiology	Comprehensive review of machine learning from basic arrhythmia mechanisms to clinical electrophysiology applications.	Findings:
			∙ ML applied across scales—from ion-channel modeling to ECG classification and atrial/ventricular mapping
			∙ Demonstrated expert-level performance in classifying AF propagation patterns on intracardiac mapping
			Gaps:
			∙ Most ML tools remain at research stage
			∙ Limited clinical integration and real-world deployment
Jone *et al*., 2022 [10]	AI in CHD	State-of-the-art “call to action” outlining AI applications in paediatric and adult CHD, and priorities for deployment.	Findings:
			∙ AI opportunities span prenatal screening, postnatal diagnosis, imaging, surgical planning, and outcome prediction
			∙ Early successes include CNN-based fetal ultrasound models for CHD detection
			Gaps:
			∙ CHD datasets are small, fragmented, and heterogeneous
			∙ Implementation barriers include lack of explainability, legal concerns, and need for clinician training
Mohammadi *et al*., 2024 [9]	AI for CHD Surgical Outcomes	Systematic review of 35 studies using AI to predict post-operative outcomes in CHD surgery.	Findings:
			∙ AI models predicted ICU stay, complications, and mortality with AUCs up to ~0.99
			∙ Outperformed conventional risk scores in multiple studies
			Gaps:
			∙ Majority of studies were retrospective
			∙ Few models underwent external validation; clinical utility yet to be proven

ML, machine learning; AI, artificial intelligence; CHD, congenital heart disease; ICU, intensive care unit; AUC, area under the curve; AF, atrial fibrillation; CNN, convolutional neural network; ECG, electrocardiogram.

## 2. AI in Diagnostics of ACHD

Precise CHD diagnosis and assessment in adults typically involve the integration of various imaging modalities, electrocardiography, and clinical information. Nowadays, AI technologies are developing to aid clinicians in every phase of the diagnostic process with enhanced detection and description of congenital anomalies and related complications. Defining advanced cardiac imaging as instrumental in ACHD management, detailed anatomic and functional data are acquired [12]. The effort to harness these resources has been increasingly supported by AI-assisted image analysis through DL models, where these can be trained on common cardiac imaging data sets and detect or classify anomalies automatically to assist clinicians with disease assessment [13]. For instance, convolutional neural networks (CNNs) (a DL model designed to recognise image patterns) are used in echocardiographic images to detect subtle CHD. One study separated patients with transposition of the great arteries (following surgery) and congenitally corrected transposition from controls on echocardiographic views with an accuracy of 98% using a CNN model [14, 15]. This demonstrates the ability of AI to identify subtle anatomic signals in images that less skilled human observers may overlook. DL has also been utilised to enable the automation of cardiac measurements: An AI program capable of estimating left ventricular ejection fraction from echocardiography without endocardial manual tracing was developed by Zhang *et al*. [16]. The AI learned to emulate expert readers, generating rapid quantitative results amenable to standardisation across providers [16]. Similarly, AI algorithms can identify valve malformations or dysfunction on imaging, for example, by detecting irregular leaflet motion, thereby facilitating earlier diagnoses of conditions such as congenital valve disease.

AI image segmentation in cardiac magnetic resonance (CMR) and computed tomography (CT) also facilitates faster processing of complex analysis of ACHD anatomy. ML algorithms automatically detect and delineate cardiovascular structures (vessels, defects, chambers) in three-dimensional (3D) image datasets and provide measures of volumes, ejection fraction, and defect size [17].

Significantly, AI technologies are reaching near-expert-level performance in analysing complex scans. One recent 2023 study developed an AI system to diagnose CHD from CT images, including 17 categories of CHD. The system was ~97% accurate in its diagnoses, matching human performance by experienced radiologists [18]. It could even create three-dimensional images of the heart based on CT data to assist cardiologists and surgeons in intuitively grasping a patient’s anatomy. These abilities demonstrate how AI can complement imaging professionals by enabling them to identify anomalies more rapidly or provide second opinions when in doubt.

One such promising strategy is applying AI to combine multimodality images seen in another type of DL model, generative adversarial networks (GANs), have been used to register preoperative CT images with intraoperative echocardiographic images in congenital heart surgery, improving surgeons’ real-time guidance. This form of image fusion, driven by AI, has the potential to enhance navigation in complex catheter-based or surgical procedures by providing a comprehensive view of septal defects or outflow tract obstructions, for example. Initial experiments also imply that AI algorithms initially trained on non-CHD images can be transfer-applied to ACHD with limited extra data [10]. Tandon *et al*. [19] demonstrated that a CNN model used in CMR analysis developed in normal hearts may be transfer-trained to analyse repaired Tetralogy of Fallot (TOF) with comparatively few CHD-specific training cases. This suggests that broad cardiology AI tools (e.g., chamber segmentation or flow measurement) can be repurposed for ACHD, thereby accelerating the development of robust diagnostic assistance tools. Different AI architectures exhibit varying strengths depending on the imaging modality and diagnostic task. CNNs are particularly effective for pattern recognition and classification tasks, such as anomaly detection in echocardiographic views, where their ability to extract spatial features makes them ideal for interpreting two-dimensional grayscale images. For example, CNNs have demonstrated high accuracy (~98%) in distinguishing postoperative transposition variants based on echocardiographic frames [14, 15].

On the other hand, GANs—a class of generative models—excel at image synthesis, registration, and enhancement, making them suitable for tasks requiring multimodal fusion or synthetic image generation. In the ACHD context, GANs have been successfully employed to align intraoperative echocardiography with preoperative CT datasets, thereby enabling real-time image fusion for surgical navigation [20, 21]. While CNNs provide rapid and accurate classification, GANs contribute to image realism and spatial alignment, which are crucial in complex anatomical visualisation. However, GANs generally require more computational resources and are sensitive to data imbalance and training instability. In contrast, CNNs are more mature and widely validated in clinical settings but may underperform when dealing with multimodal or 3D volumetric data unless they are appropriately adapted. Future diagnostic pipelines may benefit from hybrid architectures that combine the localisation precision of CNNs with the data synthesis and fusion capabilities of GANs.

## 3. Electrocardiography and Arrhythmia Detection and Outcome Prediction

ECGs are still essential in ACHD diagnostics and monitoring since many of these adult survivors are predisposed to arrhythmias (e.g., atrial arrhythmias following Fontan palliation, ventricular arrhythmias following repair of Tetralogy, etc.). AI algorithms performed very well in ECG analysis and are increasingly applied to CHD. One model based on DL detected the incidence of an atrial septal defect (ASD) by analysing a routine 12-lead ECG [22]. The model could detect subtle electrocardiographic features, such as right bundle branch block or axis deviations, associated with an unsuspected atrial septal defect through training on ASD-labelled and normal patient data. Such an instrument may be used to screen adults whose congenital defect was not recognised in childhood or to follow known ACHD patients with changes indicative of hemodynamic changes (e.g., development of right heart enlargement on ECG).

**Fig. 1.**
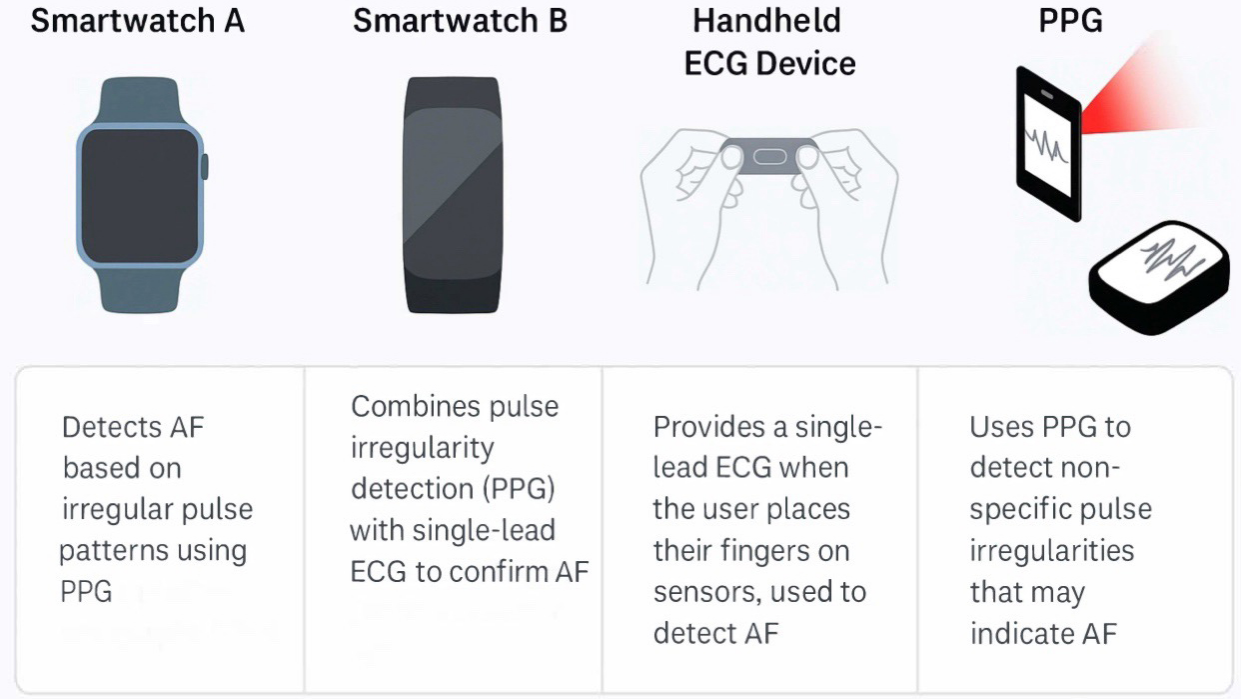
**Some of the market’s most-used wearable devices to diagnose arrhythmias in adult congenital heart disease patients**. PPG, photoplethysmography; AF, atrial fibrillation; ECG, electrocardiogram.

Aside from structural lesion diagnosis, AI also provides robust tools for arrhythmia detection and prediction. ML algorithms can automatically classify cardiac rhythms from telemetry or ECG data, as already implemented in commercially available products to detect atrial fibrillation (AF). This technology can also benefit ACHD patients with sporadic arrhythmias. Wearable (patch, watch) devices with AI algorithms have detected arrhythmias in diverse populations (Fig. 1) [23, 24].

AI may even identify ACHD patients who are at risk of developing malignant arrhythmias. Late ventricular tachycardia and sudden death are concerns in repaired TOF, and researchers have created machine-learning algorithms stratifying TOF patients as low, medium, or high risk for malignant ventricular arrhythmias based on clinical parameters. Other DL models have incorporated CMR image features to identify TOF patients who are likely to suffer from life-threatening ventricular tachycardia or cardiac arrest. With validation, these predictive models may forewarn clinicians about patients requiring more intensive rhythm monitoring or preventive interventions (e.g., placement of an intracardiac defibrillator [ICD]) [25].

Overall, by analysing more information from the ECG than a human eye can, AI-based analysis yields a type of “digital biomarker”, e.g., an AI estimate of patient-specific ECG “age” or other latent characteristics associated with ACHD outcomes, thereby enhancing risk assessment [26].

## 4. AI in Clinical Management and Therapy of ACHD

Besides risk prediction and diagnosis, AI is also used to manage and treat patients with ACHD. From planning advanced surgical procedures to medical therapy planning and follow-up, AI tools are positioned to aid clinical decision-making and maximise patient outcomes [27]. Numerous patients with CHD need one or more cardiac procedures or catheter-based interventions throughout their lifespan, frequently with anatomically complicated repairs. AI tools can assist in preprocedural planning by generating patient-specific models and simulating procedures to aid interventional cardiologists or surgeons. One of AI’s earliest and exemplary applications is 3D anatomical reconstruction. A CT or magnetic resonance imaging (MRI) of an ACHD patient can become a highly accurate 3D representation of their heart and vessels through sophisticated image segmentation. They enable clinicians to visualise aberrant connections (e.g., baffle atrial switch or conduit in place) and even simulate the intended operation in advance [28].

**Fig. 2.**
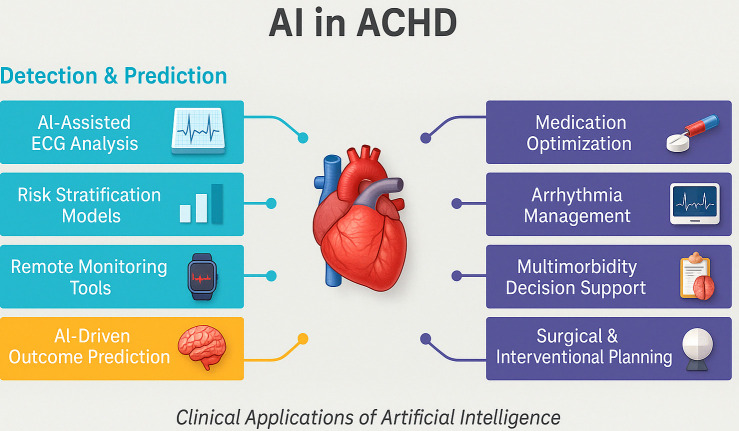
**Summary of the role of artificial intelligence in managing adult congenital heart disease**. AI, artificial intelligence; ECG, electrocardiogram; ACHD, adult congenital heart disease.

For example, before repairing a complex anomaly like total anomalous pulmonary venous return (TAPVR) in an adult patient, an AI can automatically segment the pulmonary veins and left atrium on CT, creating a straightforward map to take to the surgeon [29, 30]. This data will prove vital in determining surgery strategy and can be transferred into virtual reality spaces where the repair can be practised in advance. Incorporating anatomic models derived by AI into surgery planning can minimise theatre surprises and shorten cardiopulmonary bypass times by enabling accurate preprocedural tailoring of patch size and conduit length. Real-world clinical evaluations have begun to quantify the impact of AI-assisted 3D planning. For example, super-flexible 3D-printed heart models derived from patient CT scans were rated “essential” by surgeons in 68.4% of complex CHD cases, with accuracy validated against intraoperative findings and no reported complications, highlighting their utility in pre-surgical rehearsal [31]. In structural heart interventions, enhanced imaging fusion and 3D planning have consistently led to shorter cardiopulmonary bypass times, improved device sizing, and fewer unforeseen intraoperative adjustments. One institutional report showed a 23% reduction in operative duration and measurable decreases in procedural complications [28]. These early findings suggest that as AI-generated models and image-fusion tools become more integrated into ACHD workflows, similar efficiency and safety gains are likely, particularly in complex reoperative settings and rare anatomical variants. Summary of AI use in ACHD is demonstrated in Fig. 2.

AI can also aid during the intervention itself. As noted above, a group utilised a GAN-based solution to combine preoperative CT images with real-time transesophageal echocardiography views in the theatre [32]. The result is real-time augmented reality visualisation guiding the surgeon to structures that are too difficult to visualise directly. For catheter procedures in ACHD (e.g., stenting a coarctation or closing a residually persisting ventricular septal defect VSD), comparable AI-assisted image registration may enhance device accuracy and shorten procedure duration.

Another new frontier is computational simulation to predict procedural success: scientists are applying AI to simulate blood flow or stress distribution after a potential intervention. Computational models (with ML-aided optimisation) have, for example, been used to analyse various stent geometries in large-vessel congenital anomalies to simulate what design will reduce complications such as obstruction or thrombosis [33]. Although this strategy remains experimental, it heralds a time when surgeons may refer to AI simulators to determine the safest and most appropriate surgical approach for each unique ACHD patient.

One of the real-world impediments to practising AI broadly in planning is the expertise and time needed to create models. Developing high-fidelity patient-specific simulations can be time-consuming and may conflict with tight clinical schedules. As improving AI algorithms and computational resources become increasingly accessible, the initial burden of time should diminish. The greatest possibilities that AI-assisted planning presents, including more accurate repairs and possibly enhanced postoperative results, must be weighed against practical considerations. Initial experience suggests that dedicating time to AI-generated planning is valuable, especially in high-risk, anatomically challenging situations, where even subtle technical improvements can lead to improved patient outcomes or reduced reoperations [33]. While reinforcement learning (RL) offers a promising framework for dynamic treatment optimisation—by learning personalised strategies from longitudinal patient data—its application to ACHD presents unique challenges. Unlike conventional heart failure populations, ACHD patients often exhibit non-standard haemodynamics, residual shunts, or surgically altered physiology (e.g., Fontan or Mustard circulation), which are not adequately represented in RL training datasets derived from acquired cardiovascular disease. Furthermore, many pharmacological therapies used in ACHD are off-label, and clinical practice varies widely due to the absence of robust evidence-based guidelines. These factors create data heterogeneity and inconsistency in outcome labelling, which can impair model convergence and generalisability.

Additionally, structured electronic data on medication timing, dosage adjustments, and response in ACHD are often lacking, limiting the ability of RL agents to learn from observed trajectories. To overcome these barriers, future RL applications in ACHD may require synthetic data augmentation, incorporation of physiology-based models, and domain adaptation strategies that allow transfer learning from standard populations while adjusting for congenital-specific factors. Interdisciplinary collaboration will also be essential to define appropriate action spaces and reward functions that reflect ACHD care goals (e.g., balancing oxygenation, arrhythmia risk, and ventricular loading conditions).

## 5. Predicting Complications and Guiding Interventions

AI’s capacity to predict clinical events can have a direct impact on therapeutic planning in ACHD. For example, suppose the ML model identifies a patient with repaired Tetralogy as having a high risk of sudden cardiac death in 5 years [25]. The treatment team may choose an ICD as a preventative device or pre-emptive pulmonary valve replacement to relieve stress on the right ventricle.

Several AI algorithms targeting ACHD complications are nearing clinical usefulness. One model predicts heart failure hospitalisation in adults with complex CHD based on clinical and imaging inputs and identifies high-risk patients who can benefit from optimising heart failure drugs or advanced therapies before decompensation [20, 34]. Another model is noted as predicting stroke among ACHD patients based on data from health records and imaging. It may trigger clinicians to initiate anticoagulation or treat atrial arrhythmias more intensively in a patient identified as high risk for thromboembolism [35].

AI may also aid in the optimisation of therapy in medical management. For instance, in adults with CHD and heart failure, there may be uncertainty about how aggressively to titrate usual heart failure medications due to this atypical cardiac anatomy. Decision support by an AI system could review similar past patients and recommend an optimal dosing regimen associated with better results in those patients. With its ability to categorise patients into subgroups based on genotype or phenotype, AI may facilitate individualised treatment—for example, determining in Fontan patients those with a physiology more amenable to renin-angiotensin blockade vs those who will require early transplant referral.

Medication optimisation through AI already has applications in other parts of cardiology (e.g., using reinforcement learning to manage the dosing of blood pressure medications), and extending this to ACHD is a natural next step. Additionally, AI can prevent complications by ensuring guided care. Decision-making algorithms may prompt clinicians to recall prophylaxis precautions (e.g., endocarditis prophylaxis or thromboprophylaxis in cyanotic patients) by checking electronic health records for ACHD-specific risks. One such example was where an AI system analysed clinical notes and automatically flagged ACHD patients with prior atrial arrhythmia and a shunt and prompted a reminder of stroke prevention measures [35]. By acting as a safety net, AI can mitigate the risk of human error, resulting in fewer avoidable complications.

## 6. Long-Term Monitoring and Follow-Up

ACHD requires continuous monitoring throughout the patient’s lifetime via regular clinical evaluations and diagnostic imaging. Modern telemedicine and remote monitoring devices have created an opportunity for AI systems to track patient status continuously. ACHD patients can track their heart health using wearable sensors, smartphone apps, and home devices, which measure heart rate, rhythm, blood pressure, oxygen saturation, and activity levels. Real-time analysis of collected data streams by AI algorithms enables early identification of potential problems. An AI model that monitors patients with Fontan circulation would identify minor changes in oxygen saturation or step count patterns over weeks to detect possible heart failure or arrhythmia before automatically notifying the healthcare providers. Researchers predict that connected intelligence systems will integrate AI functions into wearable devices to provide risk assessment and execute prompt medical interventions. The implementation of this system could lead to a reduction in emergency hospital visits, as patients would receive evaluations and treatment adjustments as soon as the algorithm detects signs of deterioration. Telemedicine has become a vital healthcare method for ACHD patients who face challenges reaching specialised medical centres. AI systems enhance telehealth by processing data obtained through virtual visits and remote testing procedures. The AI-powered handheld echocardiography system enables patients at home or local providers to capture images which automatically detect major functional changes (e.g., ventricular function decline) for remote assessment by ACHD specialists. The combination of telemedicine platforms and AI analytics provides individualised follow-up care, enhancing patient engagement and improving healthcare access for ACHD patients residing in remote locations. Implementing AI in long-term care systems faces multiple challenges during its adoption process. Wearable device data often contains errors or missing information, so algorithms need thorough validation to prevent false alarms from disturbing healthcare providers and their patients. Patients’ willingness to participate in continuous monitoring varies, and privacy concerns arise when medical organisations collect personal health data from their homes. The initial research demonstrates positive results because ML models use home-based patient data, including blood pressure readings and weight measurements, to forecast impending heart failure decompensation. AI follow-up technology has the potential to transform ACHD care through continuous patient monitoring, enabling proactive medical interventions rather than reactive responses. Despite the promise of AI-enabled remote monitoring, real-world implementation faces two key challenges. First, the robustness of AI algorithms when handling noisy or incomplete wearable data is crucial. A systematic review found that the accuracy of arrhythmia-detection algorithms can drop by over 15–20% on raw, patient-collected ECG/PPG data due to motion artefacts and signal loss [35]. To address this, recent AI models incorporate signal-quality assessments, data imputation techniques, and ensemble learning to improve resilience.

Second, patient adherence has a significant impact on the efficacy of monitoring. Longitudinal studies of ICD and heart-failure remote monitoring report adherence rates ranging from 50% to 80% at 6–12 months. Predictors of higher adherence include user-friendly design, minimal alert fatigue, and perceived clinical benefit. ACHD patients, often younger and tech-savvy, may show better adherence, but dedicated studies are needed. Future research should emphasise real-world dataset validation, adherence-enhancement strategies, and integration of AI pipelines into clinician workflows to ensure that remote monitoring delivers its intended benefits.

## 7. Advantages of AI Integration in ACHD Care 

The clinical community finds AI useful for ACHD because it promises improved decision quality and better treatment outcomes. The primary advantage of AI technology is its enhanced diagnostic accuracy, combined with reduced variability in results. AI algorithms operate with perfect precision when processing images or data; thus, they may decrease missed diagnoses. AI technology delivered CT interpretation results comparable to those of senior radiologists [36]. This capability results in dependable tracking of disease progression.

AI facilitates both earlier identification of health issues and swift intervention actions. ML algorithms detect subtle heart dysfunction and arrhythmias, enabling medical staff to take proactive actions such as medication adjustments or surgical planning before clinical deterioration occurs. ACHD patients could benefit from AI monitoring that identifies problems that standard check-ups usually miss. The implementation of AI technology has the potential to boost operational efficiency while reducing time demands on clinical personnel. AI systems that process echocardiograms and MRIs enable cardiologists to eliminate routine contour tracing tasks, thus allowing them to concentrate on clinical evaluation. AI systems that generate patient history summaries, current status, and risk predictions expedite clinic visits, particularly for complex ACHD cases with extensive medical records. Implementing these tools by overloaded ACHD clinics enables them to handle growing patient numbers and reduce physician workload without compromising healthcare standards.

Patients with complex ACHD commonly receive their care from general cardiologists who may lack an extensive understanding of CHD. The AI decision support system enables general practitioners to treat patients with congenital conditions through condition-specific guidance. An AI system would notify general cardiologists about adults with a Mustard repair history of transposition who face risks of baffle obstruction or arrhythmia, thus helping them initiate suitable evaluations. According to research, obtaining enough clinical expertise for the diverse CHD population is difficult. Still, AI tools can enable customised expert-level recommendations for all patients. Such benefits lead to broader access to treatment alongside improved treatment fairness. Another benefit is personalisation, AI can help tailor care to each patient’s unique risk profile. The AI-estimated risk level of a complication will enable patients to participate in decisions about preventive interventions.

## 8. Limitations and Barriers

The application of AI in ACHD practice faces several significant limitations and obstacles that hinder its adoption. A major problem exists because high-quality data needs to be available. ML models require extensive, representative datasets for training; however, ACHD comprises numerous rare defect subtypes, and most data remain confined to individual facilities. The lack of standardisation in record-keeping, imaging protocols or patient populations between institutions makes data integration across centres challenging [37]. Existing datasets contain biases, such as excessive records from specific ethnic or geographic groups, which reduces the accuracy of AI tools for minority populations (algorithmic bias). The ongoing challenge of obtaining sufficient, diverse, and plentiful data presents a significant obstacle, as AI models can perform excellently at one hospital but fail to reproduce their success at other hospitals. The complex and diverse nature of ACHD data presents obstacles that traditional AI methods struggle to overcome. Whereas conditions like myocardial infarction exhibit relatively uniform pathophysiology and are well-represented in large, standardised datasets, ACHD encompasses highly heterogeneous anatomies and varied surgical histories, which pose unique challenges for AI algorithm development and generalisability. The eclectic nature of patient cases requires modifications to standard off-the-shelf AI algorithms. ACHD benefits from AI techniques that are generally applicable in cardiology, but often require specialised methods to process the unique patterns of residual lesions and their sequelae. The system needs modifications or synthetic training to segment ventricles from hearts that have undergone a Fontan operation, since these hearts present unique challenges for standard algorithms. The combination of small dataset size and irregular anatomical characteristics prompted researchers to develop synthetic imaging data generation and transfer learning approaches. However, these approaches create complex challenges that often yield imperfect results. The same factors that challenge humans during ACHD care create obstacles for AI models.

The reliability and validation of AI tools represent a significant point of concern. The AI models developed for ACHD analysis use retrospective data from existing studies for their testing. The systems achieve remarkable accuracy within controlled laboratory tests, but their performance remains unknown when deployed in future clinical practice. The system lacks sufficient external validation because the CNN that achieved 98% accuracy in CHD echo detection has not been proven successful with fresh patient samples outside its original research population. The absence of thorough validation processes, particularly across various centres and patient groups, makes clinicians hesitant to accept AI suggestions. The problem of model transparency exists because high-performing algorithms, including deep neural networks, operate as “black boxes”, failing to provide explanations for their decision-making processes.

Medical professionals may resist acting on predictive outputs because they cannot understand the reasoning methods used in the predictions (e.g., “this patient has an 85% chance of needing surgery within a year”). User confidence will increase when methods deliver explanations through salient image regions and key variables that influence predictions. Implementing AI into current clinical procedures presents a real-world operational challenge that must not be dismissed [38]. For AI to be useful, it must be integrated seamlessly into current systems (EHRS, imaging software) and function within the time constraints of clinic and hospital processes. Medical staff will resist adopting additional software when it necessitates manual data entry or multiple software transitions. The successful implementation of AI depends on integrating it into existing medical tools that clinicians already use, such as echocardiography machines that measure strain or electronic health record (EHR) systems that alert staff to high-risk patients. Developers should focus on creating systems that combine user-friendly design with smooth workflow integration. A knowledge deficit exists regarding AI, as numerous medical practitioners lack training in AI and exhibit reluctance when working with AI-generated results. It will be essential to educate clinicians about AI so that they can utilise it effectively, since this is a significant barrier to adoption. It is only right that the clinician feels sceptical, given that the decisions are critical for patient care. For AI to be incorporated into ACHD care, it must gain acceptance from practitioners who will use it and be ethically sound. Cardiologists may doubt whether an algorithm can account for a particular patient’s specific needs as an experienced doctor can. Trust can be gained if AI tools are proven useful in the decision-making process rather than being used as a replacement for physicians. To increase clinician acceptance, one can utilise AI as an assistant tool, for instance, by presenting an AI risk score alongside a conventional assessment, allowing clinicians to monitor the AI’s performance. As positive experiences accumulate, for example, “this tool has helped me identify several patients in my practice who had adverse outcomes”, acceptance will improve. This is where explainable AI comes into practice, as AI outputs must be explained to increase user confidence. For example, in the stroke risk model, the clinician is provided with a list of the top factors contributing to the patient’s risk, as determined by SHAP analysis, making the prediction more understandable and actionable.

The use of AI in medicine raises ethical concerns regarding accountability, bias, and patient consent. In ACHD, data security and privacy are crucial, as patients have medical records from childhood and throughout their lives. AI development involves utilising such data, which necessitates strict de-identification and security protocols to prevent data breaches. This is another concern: if the training data consisted of data from high-income country centres, the developed model may not perform well for patients in other contexts and may even exacerbate existing inequities. Fairness testing and the inclusion of diverse datasets are necessary to mitigate this issue. This is why there is also an ethical requirement for transparency; patients and providers should be informed when AI is used in their care and have the right to understand how it works.

Furthermore, human supervision is compulsory; there is an agreement among regulators and ethicists that decisions should not be made solely by AI without the clinician’s input. Regulatory bodies are initiating measures to address these challenges. It is worth noting that the European Union has introduced the AI Act (adopted in 2024), which guarantees the trustworthy, safe, and transparent use of AI systems in healthcare. This entails explaining how an AI was trained and tested, as well as its limitations. AI tools for ACHD may require formal approval by the Food and Drugs Association or European authorities before being used clinically. This process will also ensure that only validated and high-quality algorithms are deployed. On the legal side, questions of liability arise: if an AI system fails to predict a complication that occurs or, worse, if it leads to a harmful intervention, who is to blame—the doctor, the hospital, or the AI system’s producer? Future guidelines are needed to help clinicians utilise AI without incurring legal consequences and to protect patients’ rights. Beyond general concerns of privacy and transparency, AI adoption in ACHD raises unique ethical dilemmas due to the lifelong nature of care. One such issue involves the transition from paediatric to adult cardiology, a period often marked by shifts in autonomy, care engagement, and risk perception. AI-generated risk stratification or treatment suggestions made during adolescence may continue to shape decision-making into adulthood, raising questions about how evolving patient preferences are integrated into AI-informed care. Additionally, concerns exist regarding the division of responsibility between clinicians and AI. In a population with rare and surgically modified anatomies, the balance between clinical intuition and algorithmic recommendation is delicate. For example, should an ACHD provider follow an AI tool suggesting low arrhythmic risk if it contradicts the provider’s concern based on scar burden or family history? These tensions raise medico-legal and ethical questions about accountability, particularly when AI systems are embedded in care pathways. To address this, future frameworks should prioritise explainable AI, ensure human-in-the-loop governance, and involve ACHD patients in shared decision-making, especially when navigating high-stakes choices like ICD implantation or surgical re-intervention.

Algorithmic bias is a critical issue that arises when AI models are trained on non-representative datasets, leading to reduced accuracy or misclassification in underrepresented groups. Although specific analyses of bias in ACHD AI models are scarce, evidence from broader cardiovascular research highlights its significance. For instance, a large-scale evaluation of an AI-based ECG algorithm revealed that the prediction accuracy for left ventricular dysfunction varied significantly across racial groups, with reduced performance in Black patients compared to White patients, due to sampling imbalance in the training data [38]. Similarly, machine learning models predicting cardiac arrest risk have underperformed in Hispanic and Asian populations when trained primarily on White-majority cohorts [39]. In congenital cardiology, a recent analysis revealed that AI models used for subcutaneous-intracardiac defibrillator eligibility prediction in ACHD performed suboptimally in patients of African and Middle Eastern origin, prompting a call for more geographically diverse training datasets. These findings underline the risk of perpetuating healthcare disparities through biased AI tools and underscore the need for international, multiethnic datasets in ACHD AI development.

In conclusion, while AI can potentially enhance ACHD management, its application in real-life scenarios should be undertaken with caution, considering factors such as data ethics, regulatory issues, and the involvement of clinicians and patients. With proper supervision, expediting, and user education, most of these problems can be resolved, allowing AI to benefit congenital cardiology.

## 9. Future Directions and Research Gaps

ACHD and AI research is a rapidly developing field, and there are many ways to grow and improve these applications in the future. Achieving the full potential of AI in ACHD will necessitate specific research and collaboration to address current barriers. Below, we outline some key future directions and gaps that require attention. The first and most important step is the generation of large, multi-centre ACHD datasets that cover the range of CHD and patient characteristics. Data from rare CHD subtypes and outcomes will require international collaboration to be incorporated into the data pool [40]. Developing common data repositories or registries for ACHD will be useful for AI research. The quantity of the data is important. However, the data quality is crucial—this includes definitions, protocols, and follow-up data to make AI predictions more reliable. Real data can be supplemented by data augmentation and synthetic data generation. For example, generating anatomically plausible virtual MRIs has been used to enlarge training sets for Tetralogy of Fallot imaging analysis. However, synthetic data must be used carefully and only validated against real-world cases. A federated learning approach (where AI models are trained on data from multiple institutions without centralising all the data) could be a promising way to develop robust ACHD AI models while protecting patient privacy.

### 9.1 Enhancing AI Explainability and Transparency

The clinical adoption of future AI tools will be improved by incorporating explainability as a design requirement. This means moving beyond black-box predictions to provide clinicians with outputs they can understand. Explainable AI methods, such as heatmaps on imaging that show what anatomical features influenced a CNN diagnosis or summary dashboards that highlight the patient features driving a risk score, are relevant for ACHD applications. Using SHAP values in the stroke risk model is an example of incorporating explainability, but more user-friendly approaches are needed [41]. Developing ACHD-specific explainability metrics (e.g., identifying which clinical variables or imaging views are most predictive of a given outcome) will increase trust. It may also provide new medical insights into disease mechanisms.

### 9.2 Prospective Clinical Trials of AI Interventions 

The current literature has a significant gap in the absence of prospective trials on the use of AI in ACHD care. Most studies report on the development and validation of algorithms using retrospective data. The next step is to test these AI tools in clinical practice—for example, a trial where half of the ACHD clinics receive an AI risk prediction tool and the other half continue with standard care, to determine if the outcomes differ (for instance, better or earlier interventions, fewer adverse events, etc.). Prospective human validation is essential to decide whether AI adds value beyond current care models. This includes examining workflow integration: Does the AI save time or reduce errors? Such studies will also help identify unintended consequences or user errors associated with the use of AI. General cardiology has examples, such as AI-guided ECG screening studies, that can serve as templates for ACHD-specific trials. Regulators and guideline bodies will likely require evidence from these trials before recommending AI-assisted care.

### 9.3 Federated Learning (FL) 

A promising approach to overcoming data-sharing barriers in ACHD AI development is FL—a distributed training paradigm in which models are trained locally at participating centres and only model updates (not raw data) are shared with a central aggregator. However, the practical implementation of FL in ACHD imaging presents several challenges. First, there is considerable heterogeneity in imaging protocols across centres, especially in modalities such as CMR and echocardiography. Variability in acquisition parameters, segmentation standards, and data annotations must be harmonised—potentially through the adoption of standardised formats like Digital Imaging and Communications in Medicine and HL7 FHIR. Second, successful FL requires synchronous computational infrastructure, secure update protocols, and model aggregation workflows that may be infeasible for centres with limited IT resources. Third, regulatory constraints must be carefully navigated. For example, centres in the European Union must comply with the General Data Protection Regulation. At the same time, US institutions are bound by the Health Insurance Portability and Accountability Act, creating complex cross-jurisdictional requirements. Lastly, FL projects must develop evaluation metrics that track performance across sites without sharing test data and must ensure fairness auditing to prevent amplification of existing biases. Despite these challenges, the application of FL in cardiology (e.g., aortic valve segmentation, heart failure risk prediction) demonstrates its feasibility. For ACHD, federated frameworks hold significant promise for training generalisable, privacy-preserving models—especially when supported by international collaboration, common data definitions, and ethical oversight.

### 9.4 Multimodal Foundation Models for Complex Data Integration

The next generation of AI models—particularly multimodal foundation models—offers unprecedented potential to address the complexity of ACHD by integrating heterogeneous data modalities at scale. These models, pre-trained on vast corpora of unlabelled data using self-supervised learning, can be fine-tuned for ACHD-specific tasks such as risk prediction or anomaly detection. For example, a future foundation model could simultaneously ingest CMR images, 12-lead ECGs, genomic profiles, and structured and unstructured clinical data (e.g., operative notes, blood biomarkers) to generate a comprehensive, personalised risk score. This approach is particularly attractive for ACHD, where insights often depend on the interplay between structural anatomy, electrophysiology, and genetic syndromes. Moreover, these models could learn robust representations even from small ACHD datasets by leveraging cross-domain transfer from larger cardiovascular or oncology datasets. Early multimodal AI architectures (e.g., Med-PaLM, BioGPT-X) have already demonstrated impressive performance in general medicine, and similar frameworks tailored to congenital cardiology may help overcome traditional limitations of data silos and modality fragmentation. However, realising this vision will require rigorous governance around data provenance, cross-modality alignment, and interpretability, particularly given the high-stakes nature of congenital heart disease decision-making.

To bridge the gap between retrospective validation and clinical utility, prospective trials are essential to evaluate AI tools in ACHD care. Given the structure of ACHD care—often centralised in specialist centres but supported by general cardiology networks—cluster-randomised trials at the ACHD centre level could be particularly effective. For instance, centres could be randomised to implement an AI-based decision support tool (e.g., for arrhythmia risk stratification or surgical planning), while control sites continue with standard care. This design would minimise contamination and reflect real-world practice variation.

Primary endpoints in such trials might include:

• Time-to-intervention (e.g., time from risk identification to electrophysiology referral or valve surgery),

• Unplanned hospitalisations or emergency visits,

• Change in risk profile over time (e.g., serial imaging or ECG biomarkers),

• Adherence to ACHD guideline-based follow-up, and

• Patient-reported outcomes such as quality of life or perceived engagement in care.

Trials could also incorporate mixed-methods evaluations, including clinician usability feedback and health-economic analysis, to understand both effectiveness and feasibility. Ultimately, such studies would help define whether AI integration meaningfully improves decision-making, timeliness, and outcomes in this complex patient population.

### 9.5 AI for Personalised and Precision Medicine 

Future research should aim to utilise AI to tailor therapy to the individual ACHD patient. This could involve linking genomic or proteomic data with clinical data to predict risks and responses to therapies. For instance, ML might identify genetic subgroups of ACHD patients with different responses to heart failure medications or at risk of certain complications, thus enabling more personalised treatment. Precision medicine benefits from AI through its ability to make complex decisions about patient intervention times, which allows it to select the best approach from thousands of similar cases [42]. Reinforcement learning is another frontier because it enables algorithms to learn the most effective treatment policies for optimal results, including medication modifications and catheterisation timing. Future medical applications of these experimental approaches may allow clinicians to obtain evidence-based recommendations when treating chronic conditions, such as Fontan circulation failure and Eisenmenger physiology, as the optimal course of action remains uncertain.

To achieve practical success in AI development, the main focus should be on creating a seamless integration of AI systems into medical work processes. Successful integration requires electronic health record vendors and imaging software companies to develop background AI analysis capabilities that present results through the existing user interface [43]. Investigating how humans interact with computers for AI healthcare purposes will be beneficial because it will reveal which format of AI output clinicians find most effective (visual risk presentation or text notification) and strategies to prevent excessive alerts. The design and implementation of tools must involve ACHD clinicians to guarantee these solutions meet actual clinical requirements. Deploying new AI applications will require continuous frontline feedback and usage data collection to identify areas that need improvement. The ultimate goal is to create an “invisible” integration system that enables AI to function naturally during clinical decision-making, similar to modern automated lab result interpretation and dose calculation tools. AI literacy training, which teaches clinicians about the capabilities and limitations of algorithms and evaluation methods for tools, will enable them to implement these tools correctly [44]. Experts recommend establishing AI-specific certification or continuing education programs that include ACHD case studies for cardiology professionals. Patient education about AI applications in healthcare and addressing their concerns will lead to better acceptance among ACHD patients. The increasing presence of AI in healthcare will not diminish the necessity of human care for patients, so training must emphasise that AI tools support individualised, compassionate patient care.

## 10. Conclusion

AI can potentially revolutionise the ACHD field, which serves a diverse and growing patient population with complex needs. As outlined in this review, AI applications are emerging across the ACHD spectrum—from advanced imaging interpretation and automated diagnosis to risk prediction, surgical planning, and personalised long-term management. Early results are encouraging: AI algorithms can achieve high accuracy in identifying congenital disabilities and stratifying risk, offering innovative solutions for tailoring interventions and remotely monitoring patients. By leveraging modern computing power and big data, AI can refine CHD diagnosis and prognostication with greater precision, efficiency, and accessibility​. Consequently, ACHD patients stand to benefit from more timely interventions and customised care plans that account for their unique cardiac anatomy and physiology.

However, the journey toward routine clinical use of AI in ACHD is just beginning. Careful integration into clinical workflows, thorough validation in diverse patient cohorts, and ongoing attention to ethical principles will determine the ultimate success of these technologies. AI tools must be developed in partnership with clinicians and patients, ensuring they address real-world challenges and are user-friendly. Education and training will help foster a generation of clinicians comfortable using AI as a collaborative tool in decision-making. With prudent implementation, AI will not replace the physician’s expertise but rather augment it, serving as a tireless assistant that can analyse data at scale, provide evidence-based suggestions, and catch patterns that humans might overlook.

The future of ACHD care is likely to be a hybrid model in which human clinical acumen and AI work in tandem. For example, an ACHD specialist in a clinic might use an AI-generated risk report to inform a discussion with the patient about prophylactic therapies, or a multidisciplinary team might review a surgical plan aided by AI-derived 3D models and simulations. In such scenarios, the clinician’s holistic understanding of the patient, including values and preferences, remains central, while AI contributes additional insight and rigour to inform the decision. As technology and medicine continue to evolve, adult congenital cardiology is poised to benefit greatly from the AI revolution. With ongoing research and collaboration, AI-driven tools will become integral to delivering high-quality, personalised care, helping ACHD patients survive and thrive well into the future​. The future of ACHD care is likely to be a hybrid model in which human clinical acumen and AI work in tandem. For example, an ACHD specialist in a clinic might use an AI-generated risk report to inform a discussion with the patient about prophylactic therapies, or a multidisciplinary team might review a surgical plan aided by AI-derived 3D models and simulations. In such scenarios, the clinician’s holistic understanding of the patient, including values and preferences, remains central, while AI contributes additional insight and rigour to inform the decision. As technology and medicine continue to evolve, adult congenital cardiology is poised to benefit greatly from the AI revolution. With ongoing research and collaboration, AI-driven tools will become integral to delivering high-quality, personalised care, helping ACHD patients survive and thrive well into the future.

To translate this vision into clinical reality, the following top-priority actions are essential:

• Establishing large-scale, multi-centre, and interoperable ACHD datasets to improve the representativeness, robustness, and generalisability of AI models.

• Undertaking prospective clinical trials to evaluate the real-world impact of AI tools on ACHD outcomes and workflow efficiency.

• Prioritising the development of explainable AI frameworks to enhance transparency, foster clinician acceptance, and ensure ethical implementation.

Focusing on these areas will enable AI to evolve from a promising technology to a trustworthy and indispensable partner in lifelong care for CHD.
